# Unveiling genetic signatures associated with resilience to neonatal diarrhea in lambs through two GWAS approaches

**DOI:** 10.1038/s41598-024-64093-6

**Published:** 2024-06-06

**Authors:** Yalçın Yaman, Yiğit Emir Kişi, Serkan S. Şengül, Yasin Yıldırım, Veysel BAY

**Affiliations:** 1 Department of Genetics, Faculty of Veterinary Medicine, Siirt University, Siirt, 56000 Türkiye; 2 Sheep Research and Breeding Institute, Bandırma Balikesir, Türkiye; 3 Department of Animal Science, Faculty of Agriculture, Ege University, İzmir, 35100 Türkiye

**Keywords:** Neonatal diarrhea, Single-locus GWAS, Multi-locus GWAS, Genetic resilience, Genetics, Animal breeding, Genetic association study, Genetic markers

## Abstract

Neonatal diarrhea presents a significant global challenge due to its multifactorial etiology, resulting in high morbidity and mortality rates, and substantial economic losses. While molecular-level studies on genetic resilience/susceptibility to neonatal diarrhea in farm animals are scarce, prior observations indicate promising research directions. Thus, the present study utilizes two genome-wide association approaches, pKWmEB and MLM, to explore potential links between genetic variations in innate immunity and neonatal diarrhea in Karacabey Merino lambs. Analyzing 707 lambs, including 180 cases and 527 controls, revealed an overall prevalence rate of 25.5%. The pKWmEB analysis identified 13 significant SNPs exceeding the threshold of ≥ LOD 3. Moreover, MLM detected one SNP (s61781.1) in the *SLC22A8* gene (*p*-value, 1.85eE-7), which was co-detected by both methods. A McNemar’s test was conducted as the final assessment to identify whether there are any major effective markers among the detected SNPs. Results indicate that four markers—oar3_OAR1_122352257, OAR17_77709936.1, oar3_OAR18_17278638, and s61781.1—have a substantial impact on neonatal diarrhea prevalence (odds ratio: 2.03 to 3.10; statistical power: 0.88 to 0.99). Therefore, we propose the annotated genes harboring three of the associated markers, *TIAM1*, *YDJC*, and *SLC22A8*, as candidate major genes for selective breeding against neonatal diarrhea.

## Introduction

The neonatal mortality rate in livestock has exhibited a persistent lack of change over the preceding four decades, signifying a noteworthy diminution in farm revenue, exacerbating wastage, and impinging upon animal welfare. Although there has been a cumulative accumulation of scientific knowledge regarding neonatal adaptation biology after birth, its translation into tangible enhancements in survival rates remains elusive^1^. Among other factors, neonatal diarrhea, pneumonia, and sepsis stand out as leading causes of mortality, exerting a global impact on the livestock industry^2–4^. Neonatal diarrhea presents a notable challenge in farm animal management due to its complex etiopathogenesis, resulting in high morbidity and mortality rates, alongside considerable economic implications^5^. This condition exhibits a notable prevalence on a global scale and is associated with significant reductions in production efficiency among farm animals, encompassing calves, lambs, piglets, and goat kids^6^. The economic losses occur due to high morbidity rates, treatment costs, and increased mortality^7–9^. The disease is associated with changes in gut microbiota, serum immunological, and biochemical parameters, impacting the health and welfare of lambs in intensive production systems^9^. Additionally, the etiological and pathomorphological investigations of coronavirus and rotavirus gastroenteritis in goat kids and lambs have shown increased treatment costs and substantial economic losses to farmers due to retarded growth and development, high morbidity, and mortality rates^8^. Furthermore, the prevalence of multi-drug resistant *Escherichia coli* in diarrheic ruminants contributes to massive economic and productive losses in the livestock industry globally^10^. The presence of *Cryptosporidium parvum*, *Escherichia coli* K99, rotavirus, and coronavirus in neonatal lambs has been linked to high morbidity rates, leading to economic losses^11^. Additionally, neonatal diarrhea is a primary contributor to the neonatal diarrhea complex, along with pathogens such as *Cryptosporidium parvum*, rotavirus, coronavirus, and *Escherichia coli*, further highlighting its economic impact^12^.

Risk factors associated with passive immunity, health, birth weight, and growth performance in lambs have been identified, impacting morbidity and mortality rates, which are above economically acceptable levels^13^. The etiology of neonatal diarrhea is multifactorial, involving various infectious agents such as *Cryptosporidium parvum*, *Giardia* spp., *Escherichia coli*, rotavirus, and coronavirus^11,14–16^. The zoonotic potential of neonatal diarrhea in livestock is a significant public health concern due to the potential transmission of pathogens from animals to humans. Several studies have highlighted the risk of zoonotic transmission of enteric pathogens associated with neonatal diarrhea in livestock. For instance, *Giardia duodenalis*^17,18^, *Campylobacter jejuni*, *E. coli*^19^, methicillin-resistant *Staphylococcus aureus*^20^, rotaviruses^21^, and coronaviruses^22^ have been considered to be capable cross-transmission between humans and animals.

The difficulties in controlling neonatal diarrhea in livestock stem from the diverse etiology of the condition, the need for more effective preventive measures, and the variability in the efficacy of available treatments. Addressing these challenges requires a multifaceted approach that considers the complex nature of neonatal diarrhea and the need for innovative solutions to mitigate economic losses and ensure animal and public health.

While genome-wide association studies have made significant strides in uncovering genetic factors related to non-specific resilience or susceptibility to multifactorial infectious diseases caused by various pathogens like mastitis^23–25^, metritis^26,27^, and digital dermatitis^28,29^, the molecular-level exploration of genetic resilience or susceptibility to neonatal diarrhea in farm animals remains conspicuously absent in the literature. To date, no study has delved into this aspect, leaving a significant gap in our understanding of neonatal diarrhea in farm animals. Nevertheless, intriguing observations have been made indicating that despite carrying enteric pathogens, many neonatal animals exhibit no clinical signs of diarrhea^9,30,31^. These observations provide significant clues suggesting the existence of genetic resilience or susceptibility conferred by innate immunity against neonatal diarrhea, independent of infectious agents.

In genome-wide association (GWA) studies, the Mixed Linear Model (MLM) stands out as the most frequently employed statistical approach, adept at mitigating issues related to population stratification and inflation arising from polygenic backgrounds^32^. Nonetheless, the reliance on single-marker MLM-based analyses may limit the detection of multiple loci concurrently influencing the traits of interest due to its one-dimensional genome scanning approach. Consequently, this method may be inadequate for capturing polygenic effects. Furthermore, MLM-based GWA studies face challenges regarding multiple correction tests for determining statistical significance thresholds^33,34^. To tackle these challenges, the multi-locus polygenic-background-control-based Kruskal–Wallis test plus empirical Bayes (pKWmEB) method has emerged as a valuable alternative for nonparametric data. Therefore, we opted to utilize the multi-locus pKWmEB and single-locus MLM methods to leverage their respective strengths.

Finally, to determine whether there is any major-effected marker among the SNPs with detected additive effects in both GWA models and to assess the potential usability of these markers for selective breeding a McNemar's test for correlated proportions was conducted.

Thus, the work described here employed two GWA approaches: the nonparametric multi-locus method pKWmEB and the single-locus Mixed Linear Model (MLM) to investigate potential associations between variations in the genetic background of innate immunity and neonatal diarrhea in Karacabey Merino lambs. The final aim of the work is to evaluate if there is any potential to perform selective breeding using major effective markers.

## Results

The overall prevalence of diarrhea in neonatal lambs was calculated to be 25.5% (180 cases, 527 controls, with a distribution of 24.1% in males and 26.7% in females. No significant differences were found between males and females (Chi-square, 0.613; exact *p-value*, 0.434).

**Figure 1:**
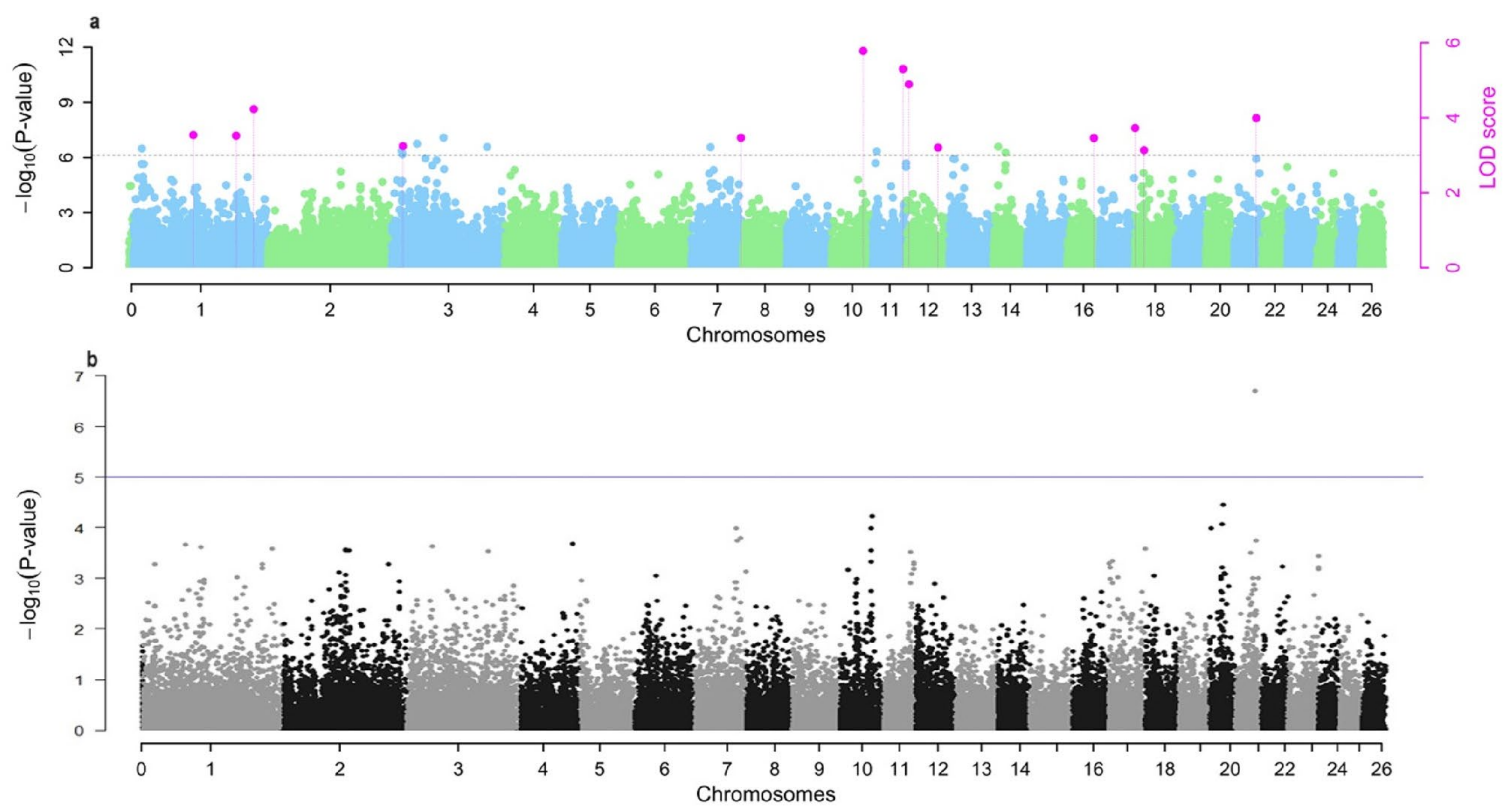
Manhattan plots for pKWmEB and MLM.

After quality controls (QC), 36,529 SNPs and 707 lambs remained for further analysis. The multi-locus approach (pKWmEB) identified 13 SNPs meeting the threshold of ≥ LOD 3 (Table 1; Fig. 1a). Within a 300 KB ± proximity of the associated SNPs, 11 known and two novel candidate genes (ENSOARG00000016287, and ENSOARG00020003584) were detected. Seven of the associated SNPs were located within the relevant gene.

**Table 1:** Identified neonatal diarrhea associated SNPs using multi-locus pKWmEB method.

CHR	Marker	Rs ID	SNP Effect	LOD score	r^2^ (%)^1^	MAF	Allele	Location	Nearest gene^2^	Distance (Kb)	Association type
1	oar3_OAR1_253469726	rs413721879	−0.0738	4.23	2.9	0.44	G	Missense variant	SLCO2A1	–	Protective
1	oar3_OAR1_122352257	rs424708132	0.0713	3.54	1.87	0.35	T	Intron	TIAM1	–	Risk
1	Chr1:212179926	na	0.0631	3.52	1.63	0.31	T	Intron	NAALADL2	–	Risk
3	oar3_OAR3_22100400	rs425892462	0.0677	3.25	1.19	0.26	T	Upstream	ENSOARG00000016287	9	Risk
7	oar3_OAR7_99600976	rs426649041	−0.0507	3.47	1.29	0.29	G	Intron	GOLM2	–	Protective
10	OAR10_67633398.1	rs414881525	0.0863	5.78	3.49	0.42	T	Intergenic	ENSOARG00020003584	239.3	Risk
11	oar3_OAR11_56555995	rs417366865	−0.0777	5.3	2.18	0.38	A	Intergenic	SDK2	73.8	Protective
12	oar3_OAR12_4667175	rs427792018	0.0768	4.89	1.17	0.26	C	Intergenic	CR2	57.7	Risk
12	s67370.1	rs412315678	−0.0004	3.2	2.3	0.5	C	Intergenic	CEP350	5.8	Protective
16	OAR16_66599845.1	rs428278614	0.0765	3.46	1.22	0.3	G	Intron	CTNND2	–	Risk
17	OAR17_77709936.1	rs422584795	0.0533	3.73	1.9	0.35	T	Intron	YDJC	–	Risk
18	oar3_OAR18_17278638	rs416409832	−0.0435	3.13	1.09	0.27	T	Intergenic	AGBL1	244.8	Protective
21	s61781.1	rs404606866	−0.0931	3.99	1.5	0.2	A	Intron	SLC22A8	–	Protective

^1^The proportion of phenotypic variance explained by significant SNP(%).

^2^Gene annotation was performed using ARS-UI_Ramb_v2.0 and Oar_v3.1 assemblies.

The classical MLM approach identified one SNP with an exact *p*-value of 1.85e-7 (Table 2, Fig. 1b). The SNP, s61781.1 in the *SLC22A8* gene, was co-detected by both methods.

**Table 2:** Identified neonatal diarrhea associated SNP using single-locus MLM method.

CHR	Marker ID	Rs ID	Beta	SE	MAF	Allele	*p-*value	-log10 (P)	Bonferroni corrected *p-*value	Association type
21	s61781.1	rs404606866	−1.56E-01	2.97E-02	0.2	A	1.85E-07	6.73	0.0066	Protective

**Figure 2:**
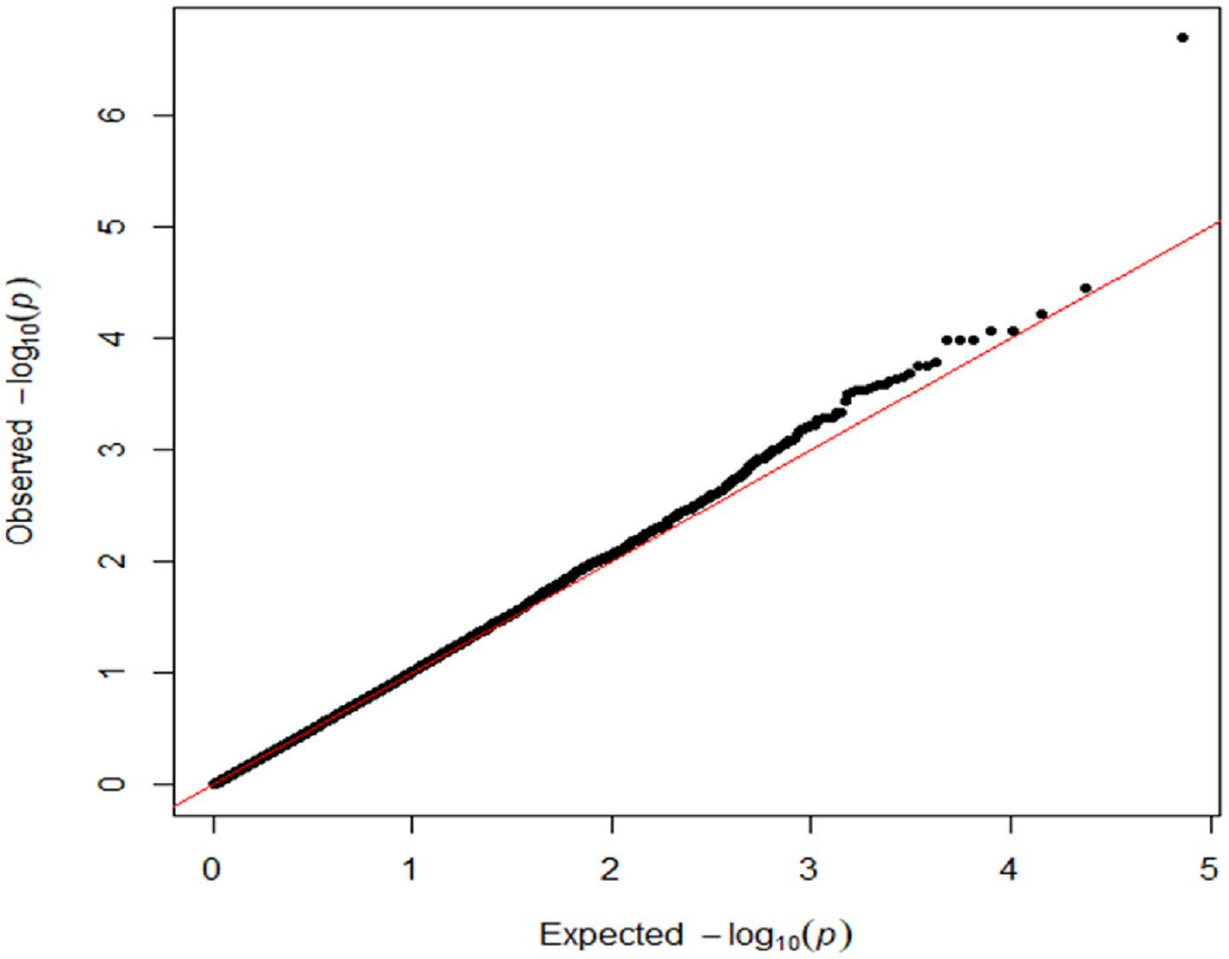
QQ plot for detecting inflation or deflation of the GWAS results.

The quantile–quantile (QQ) plot showed no significant evidence of inflation or deflation factors in the test statistics. (λ = 1.03). This confirms that potential population stratification and/or cryptic relatedness were effectively controlled during the GWA studies (Fig. 2).

**Figure 3:**
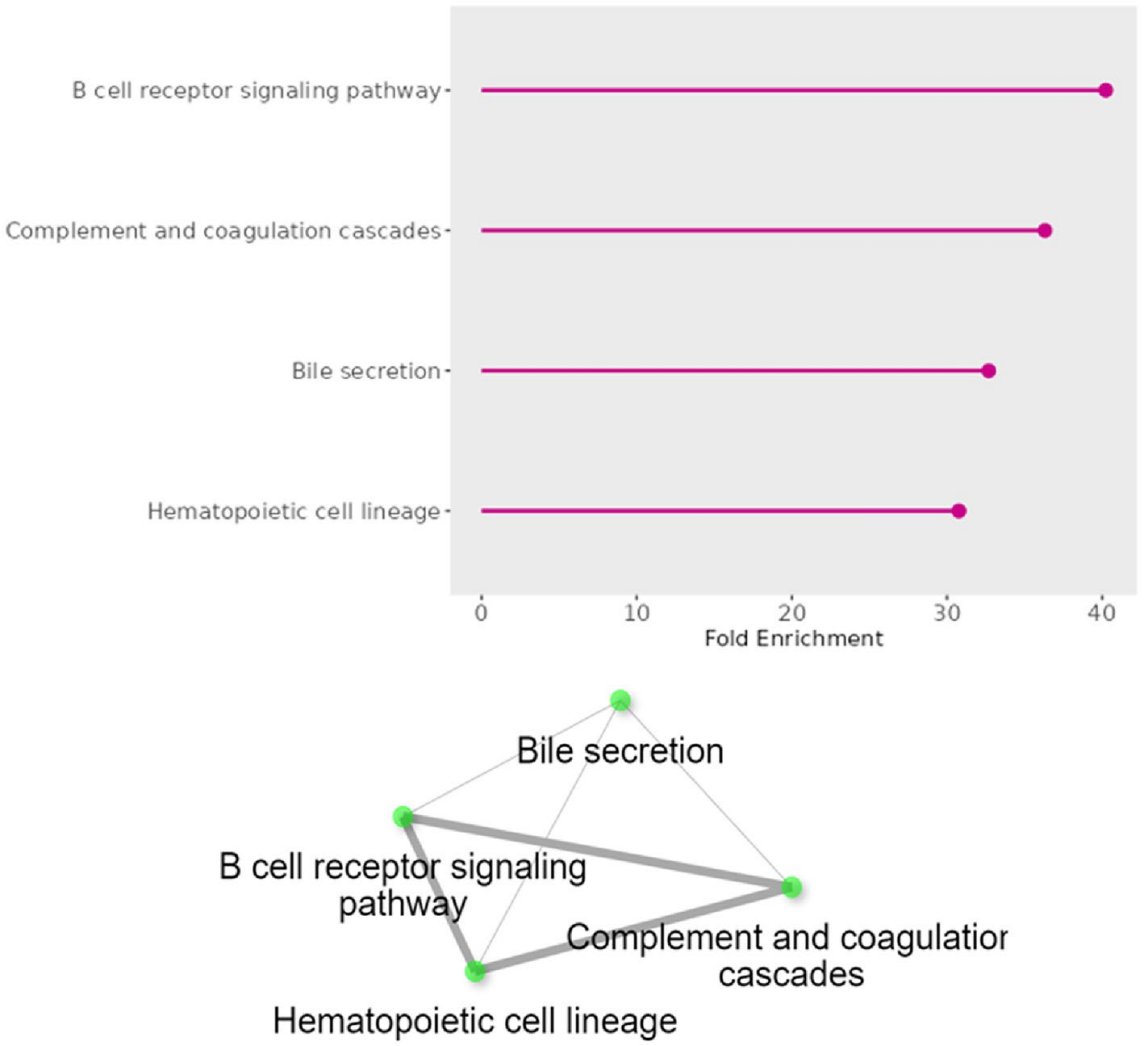
Gene enrichment and network analysis of associated candidate genes.

Gene enrichment and network analysis using the genes which are 100 KB ± distance to relevant SNP showed that identified candidate genes play a role within B cell receptor (BCR) signaling, the complement and coagulation cascade, the hematopoietic cell lineage, and the bile secretion pathways (Fig. 3).

In McNemar’s test, four SNPs (rs424708132, rs422584795, rs416409832, and rs404606866) met the statistical power critical threshold, i.e., power ≥ 0.80, and *p*-value ≤ 0.05. The achieved statistical powers were observed to range between 0.88 and 0.99. Additionally, it was noted that the odds ratios (OR) for these SNPs ranged from 2.03 to 3.10. Among these four SNPs, the first two were observed to contribute to genetic susceptibility to neonatal diarrhea, while the last two were found to confer genetic resilience. All of these SNPs with major effects were affecting the occurrence of neonatal diarrhea in the dominant model (Table 3).

**Table 3:** The results of McNemar’s statistics and statistical power of each test.

Marker	Anotated gene	McNemar pairs (case/control)					Pairs *n*	McNemar’s χ^2^	OR	Association type	McNemar* p*-value	Statistical power	Statistical power *p*-value
		+,+	+,−	−,+	−,−	Discordant pairs							
		a	b	c	d	b + c							
rs424708132	TIAM1	70	59	19	32	78	180	20.5	3.1	Risk	4.40E-06	0.99	0.02
rs422584795	YDJC	65	59	29	27	88	180	10.23	2.03	Risk	9.80E-04	0.88	0.03
rs416409832	Intergenic	41	27	57	55	84	180	10.71	2.11	Protective	7.80E-04	0.92	0.05
rs404606866	SLC22A8	20	20	54	86	74	180	15.62	2.7	Protective	6.20E-05	0.98	0.05

## Discussion

The report described here presents the genetic background of neonatal diarrhea in farm animals for the first time, utilizing the sheep paradigm. Using two GWA approaches (single-locus MLM and multi-locus pKWmEB), a total of 13 markers were identified affecting the prevalence of neonatal diarrhea with small effects ranking from 1.09% to 3.49% (Table 2). Among these 13 markers, s61781.1 (rs404606866) was co-detected by both methods with genome-wide significance. After conducting a McNemar's test for correlated proportions to investigate whether there are any markers with major effects among them, it was determined that four markers exhibit significant influence, with odds ratios ranging from 2.03 to 3.1 (statistical power ranging from 0.88 to 0.99). Once again, the marker s61781.1 (rs404606866) surpassed the third test. Three of these markers, namely oar3_OAR1_122352257, OAR17_77709936.1, and s61781.1, were found within intron regions of protein-coding genes (with rs IDs rs424708132, rs422584795, and rs404606866, respectively). The annotated genes for these three markers were identified as *TIAM1*, *YDJC*, and *SLC22A8*.

In the Sheep Breeding and Research Institute, an official organization affiliated with the Turkish Ministry of Agriculture and Forestry, which has an 80 year history in sheep farming and although it boasts considerable professional resources and personnel, the prevalence of neonatal diarrhea has been observed to reach concerning levels, such as 25.5%. As far as the authors are aware, no study has investigated the genetic factors contributing to resilience or susceptibility to neonatal diarrheas among farm animals at the molecular level. Nonetheless, there are noteworthy observations suggesting that despite harboring pathogens, many neonatal animals remain asymptomatic of diarrhea. For instance, in their case-control examination, Caffarena et al.^31^ found that while their pen mates developed diarrhea (case groups; n = 264), the control groups (n = 271) consisted of non-diarrheic calves testing positive for *Cryptosporidium* spp. (26.9%), bovine astrovirus (22.4%), Rotavirus (11.1%), *Salmonella enterica* (2.6%), or *Escherichia coli* (1.8%). In another investigation, Abdou et al.^30^ discovered that the prevalence of Group A rotaviruses in non-diarrheic cattle, sheep, and goats was 2.9, 1.3, and 2.8%, respectively. Zhong et al.^9^ provided a possibly more dramatic example when they showed that the relative prevalence of potentially pathogenic bacteria, like Bacteroides, in healthy lambs was more than double that in diarrhoeic pen mates using 16S rRNA sequencing of the gut microbiota.

These findings support the authors’ conclusions, highlighting that variations in individuals’ genetic backgrounds concerning innate immunity, coupled with other environmental factors like climatic conditions, management practices, and colostrum intake, significantly influence disease outcomes.

The present study delves into the intricate genetic landscape that underpins immunity, with a focus on key genes identified through dual GWAS. The *SLCO2A1* gene encodes a crucial prostaglandin transporter essential for the cellular uptake of Prostaglandins (PGs)^35^. These PGs play pivotal roles in chronic inflammation by amplifying cytokine effects on inflammatory cells and regulating gene expression, thereby promoting autoimmune inflammation through the differentiation and proliferation of Th1 and Th17 cells^36^. Additionally, SLCO2A1 facilitates prostaglandin E2 (PGE2) secretion by macrophages, modulating neutrophil clearance from inflammatory sites^37^.

The TIAM family proteins, encompassing TIAM1 and TIAM2/STEF, function as Rac1-specific Guanine Nucleotide Exchange Factors (GEFs), holding pivotal significance across diverse cell types, including epithelial, neuronal, and immune cells. Termed Tiam GEFs, these proteins intricately regulate essential cellular processes such as migration, proliferation, and survival by initiating and guiding Rac1 signaling pathways. Perturbations in Tiam GEFs are intricately associated with a broad spectrum of human ailments, ranging from cancer to immunological and neurological disorders^38^. *TIAM1* plays a role in the Wnt signaling pathway, engaging with proteins such as TAZ/YAP to govern gene expression and cellular functions^39^. Furthermore, *TIAM1* participates in glucose-stimulated insulin secretion within pancreatic β-cells, underscoring its involvement in metabolic activities^40^.

The N-acetylated alpha-linked acidic dipeptidase-like 2 (*NAALADL2*) gene is implicated in various molecular functions and diseases, including intestinal Behçet’s disease^41^, inflammation, apoptosis, and cardiovascular pathology in Kawasaki disease^42^. Moreover, NAALADL2 plays a role in Epstein–Barr virus infection, which is associated with disorders such as multiple sclerosis and lupus^43^.

The *GOLM2* gene, also referred to as *CASC4* and identified as a potential cancer susceptibility gene^44^, has been linked to reduced overall survival rates in ovarian cancer^45^. Recent studies conducted during the pandemic have highlighted the *GOLM* gene family, including GOLM1 and GOLM2, identifying them as potential causal plasma proteins associated with SARS-CoV-2 and suggesting their involvement in increased susceptibility to COVID-19 infection^46,47^^.^

The *SDK2* gene is associated with circulating serum soluble gp130 (sgp130), an antagonist of the inflammatory response in atherosclerosis mediated by interleukin 6^48^. The role of *SDK2* in immunity is indicated by its altered stage-specific expression during HIV-1 infection^49^. Additionally, it has been demonstrated that *SDK2* is differentially expressed upon experimental infestation by the protozoan *Amyloodinium ocellatum* in European seabass tissues^50^.

Complement receptor (CR) type 2 (CR2/CD21) exhibits expression primarily during both the immature and mature stages of B cell maturation. In conjunction with CD19, CR2 plays a pivotal role in enhancing mature B cell responses to foreign antigens^51^. Research indicates that deficiency in CR2 could alter antibody production and lead to the deposition of immune complexes, thereby influencing the humoral immune response^52^. Moreover, CR2 has been linked to the recognition of foreign DNA during host-immune responses, highlighting its role in immune surveillance and pathogen response^53^.

The *CEP350* gene has been implicated in the transcriptional response of macrophages triggered by *Mycobacterium tuberculosis*^54^ and displays distinct expression patterns in erythema nodosum leprosum^55^. Additionally, CEP350 proteins interact with viral proteins of SARS-CoV-2 and exhibit altered expression upon infection^56^.

*CTNND2* undergoes downregulation in the later stages of Cytomegalovirus infection in humans^57^. Notably, both *CEP350* and *CTNND2* genes are similarly implicated in the macrophage transcriptional response to *Mycobacterium tuberculosis*^54^.

The *YDJC* gene plays a pivotal role in autoimmune diseases by interacting with *UBE2L3* promoters and co-bound transcription factors^58^. Furthermore, the involvement of the *YDJC* gene has been observed in other diseases such as Coeliac Disease^59^, idiopathic inflammatory myopathies^60^, and autophagy-dependent intracellular pathogen defense^61^.

The significant role of the SLC superfamily in drug transportation is widely recognized. As a member of this family, *SLC22A8* encodes the protein OAT3, influx transporter protein, which primarily facilitates the uptake of substrates into cells^62^. The *SLC22A8* gene is also implicated in various biological processes, including survival and immune infiltration in clear cell renal cell carcinoma (ccRCC), a prevalent form of renal malignancy worldwide^63^. Additionally, prior evidence suggests a link between acute neuroinflammation and alterations in the levels of prostaglandin E2, a substrate of SLC22A8. This association is accompanied by changes in SLC22A8 levels^64^.

Network analysis using the KEGG database identified four main pathways. Among these, the B cell receptor (BCR) signaling pathway, the complement and coagulation cascade pathways, and the hematopoietic cell lineage pathway emerged as directly associated with immunity, playing critical roles in immune cell activation, regulation, and differentiation.

The B cell receptor (BCR) signaling pathway serves as a pivotal mechanism in orchestrating the immune response upon antigen encounter. Upon binding of antigens to the BCR, a cascade of events is initiated, beginning with the clustering of BCRs and culminating in the activation of genes crucial for B-cell function and differentiation^65^. This pathway is characterized by the rapid phosphorylation of key molecules such as Igα/β and intracellular signaling proteins like lyn, syk, and BLNK^66^. Such signaling triggers essential processes including B-cell proliferation, differentiation, and antibody production. Co-stimulatory signals provided by molecules like CD40 further potentiate the response, particularly in the proliferation of antigen-stimulated B cells. Regulation of the BCR signaling pathway is finely tuned, with molecules such as CD22 exerting inhibitory effects on IgG1 B-cell receptor signaling, thereby impacting B-cell development^67^. Continuous signaling through CD22 is crucial for the normal development and survival of B cells within lymphoid tissues (Akatsu et al., 2022). Additionally, B cells play a pivotal role in antigen presentation to CD4 + T cells, facilitating B-cell help and subsequent differentiation into memory or plasma cells. Furthermore, this pathway is implicated in the production of IL-10 by B-1 cells, thereby contributing to the regulation of immune responses^68^.

Complement, an integral aspect of the innate and acquired immune system, operates through a series of proteolytic cascades initiated upon encountering microorganisms^69^. Activation of complement triggers potent proteolytic cascades, leading to opsonization, pathogen lysis, and the induction of inflammatory responses via proinflammatory molecular production^70^. However, dysregulation of the complement system can occur in various diseases, causing the tightly controlled proteolytic cascade to become harmful^71^. The complement system serves a pivotal role in innate immunity, bridging it with acquired immunity^72^. It defends against foreign pathogens by generating complement fragments that facilitate opsonization, chemotaxis, leukocyte activation, and cytolysis^73^. Blood coagulation, another intricate cascade, involves coagulation proteins as its core components, orchestrating a series of reactions that culminate in the conversion of soluble fibrinogen to insoluble fibrin clots. At the heart of this process lies thrombin, which binds to fibrin during clot formation, thereby enhancing clot strength and stability^74,75^. Protease-activated receptors, such as those activated by thrombin, belong to the family of G protein-coupled receptors, serving as mediators of innate immunity responses^76^. Moreover, the kallikrein-kinin system represents an endogenous metabolic cascade, with its activation leading to the release of vasoactive kinins, including bradykinin-related peptides. This intricate system involves precursors known as kininogens and primarily tissue and plasma kallikreins. The pharmacologically active kinins, often regarded as either proinflammatory or cardioprotective, are implicated in numerous physiological and pathological processes^77^.

The hematopoietic cell lineage pathway governs the intricate process of blood cell development, beginning with hematopoietic stem cells (HSCs) endowed with the capacity for self-renewal or differentiation into two primary progenitors: common lymphoid progenitors (CLPs) and common myeloid progenitors (CMPs). CLPs give rise to lymphoid lineage cells, including natural killer (NK) cells, T lymphocytes, and B lymphocytes, while CMPs differentiate into myeloid lineage cells, comprising various leukocytes, erythrocytes (red blood cells), and megakaryocytes responsible for platelet production, crucial for hemostasis. Differentiating cells express distinct surface markers indicative of their stage and lineage, facilitating their identification and characterization^78,79^.

In addition, network analysis revealed the bile secretion pathway that could have an indirect association with immunity. The bile secretion pathway orchestrates a sophisticated interplay of molecular determinants and signaling cascades crucial for maintaining liver function and whole-body homeostasis. Regulation of bile formation and secretion involves a complex network of factors, both in animal models and humans^80^. Central to this process are the membrane transport systems within hepatocytes and cholangiocytes, along with the structural integrity of the biliary tree. Hepatocytes, comprising the majority of liver cells, are responsible for generating primary bile within their canaliculi^81^. Moreover, beyond their traditional role in aiding lipid digestion, bile acids (BAs) function as vital signaling molecules, influencing lipid and glucose metabolism and modulating the gut microbiota composition^82^. Hence, one could speculate that bile formation and/or the composition of gut microbiota influenced by bile secretion may impact the pathogenesis of intestinal pathogens responsible for neonatal diarrhea.

Notably, annotated genes in the study highlight their potential role in immune response modulation and the pathogenesis of various diseases. For example, annotated genes are implicated in chronic inflammation, amplifying cytokine effects on inflammatory cells, inflammation and apoptosis, susceptibility to COVID-19, stage-specific expression during HIV-1 infection, B cell maturation, B cell responses to foreign antigens, recognition of foreign DNA, transcriptional response of macrophages triggered by *Mycobacterium tuberculosis*. Similarly, the identified pathways are somehow linked to the immune system. Taken together, the findings underscore that most of the identified SNPs and annotated genes are directly or indirectly involved in the host immune response, suggesting potential roles in immune response to neonatal diarrhea.

In conclusion, our findings draw attention to the significant challenge posed by the prevalence of neonatal diarrhea and its economic implications. Addressing neonatal diarrhea in sheep farming is crucial not only for the well-being of the animals and the sustainability of the industry but also for public health and food safety considerations. This study reveals a subtle connection between neonatal diarrhea and genes intricately involved in immune system regulation.

Furthermore, the results of the final statistical analysis, the McNemar’s test, have revealed that four SNPs have a major effect on neonatal diarrhea with ≥ 0.88 statistical power (*p* ≤ 0.05). One of these SNPs is located in an intergenic region, while the other three are found in the intron regions of protein-coding genes. Therefore, we propose that these identified four SNPs, oar3_OAR1_122352257 (risk OR 3.10), OAR17_77709936.1 (risk OR 2.03), oar3_OAR18_17278638 (protective OR 2.11), and s61781.1 (protective OR 2.7), are candidate major effective markers, and the annotated three genes, *TIAM1*, *YDJC*, and *SLC22A8*, are candidate major genes for neonatal diarrhea in lambs. Thus, conducting selective breeding using risk markers for negative selection and protective markers for positive selection could result in a cumulative OR of 9.94 and potentially improve the herd’s innate immunity against neonatal diarrhea.

## Method

### Animals

The animal material utilized in the present study consisted of male (*n* = 332) and female (*n* = 375) Karacabey Merino lambs reared at the Sheep Breeding and Research Institute (SBRI). Karacabey Merino, acknowledged as one of Turkey’s most popular commercial sheep breeds, underwent improvement efforts in the 1940s by strategically backcrossing German Mutton Merino rams with native Kivircik ewes. After these breeding efforts, the Karacabey Merino population has maintained its genetic integrity without backcrossing for over four decades. The SBRI, functioning as the primary breeding center, has played a pivotal role in distributing Karacabey Merino sheep across Turkey since the 1940s.

Official veterinary records from the SBRI were obtained. There were 707 records for neonatal lamb which 180 of them experienced diarrhea in their neonatal life. Specifically, all the lambs were selected from two adjacent shelters allocated for males and females and subjected to uniform management practices. Thus, each lamb chosen for the study was considered to have an equal risk of infection, accounting for both the intensity and duration of exposure to pathogens.

### Genotyping and quality controls

The animals were subjected to genotyping using the OVINE 50 K BeadChip on the ILLUMINA platform at a private laboratory, resulting in an overall genotyping rate of 0.995. Initially, genotype data pertaining to sex chromosomes were excluded from the further analysis. Subsequently, quality control procedures were carried out using Plink 1.07^83^. The applied quality control (QC) parameters included the following criteria: Minor Allele Frequency (MAF) > 0.05, animal missing genotype rates (mind) < 0.1, and adherence to the Hardy–Weinberg equilibrium threshold > 0.00001. Upon the completion of the QC process, a total of 36,529 SNPs and 707 lambs satisfied the predefined threshold criteria.

### Association study

The GWA analyses were conducted using two approaches: the conventional single-locus MLM and the recently developed multi-locus pKWmEB. For both tests, the same parameters were used. Binary-formatted traits (diarrheic and non-diarrheic, or case-control) were derived from official veterinary records and used as traits for GWAS. Gender and birth type (single, twin, or triplet) were considered mixed effects. The quality of colostrum taken by the lamb is associated with the age of the mother and is among the major factors contributing to the onset of diarrhea. Therefore, the maternal age was also considered as a fixed effect. Lastly, to address population stratification, Principal Component Analysis (PCA) was conducted using TASSEL software^84^, and the first five principal components were included in the both statistical models.

pKWmEB GWA analysis was performed using the default parameters with mrMLM, an R package^85^. Kinship matrices were generated via the mrMLM software and incorporated into the model to control for cryptic relatedness. In multi-locus GWA studies, a generally accepted threshold for the logarithm of odds (LOD) score is LOD ≥ 3^33,34,86,87^. Thus, we employed the threshold LOD ≥ 3 for statistical significance of the pKWmEB method.

The single-locus MLM model was conducted using GEMMA software^88^. Kinship matrices were generated with the GEMMA for this test. The Bonferroni correction was applied for genome-wide statistical significance.

Manhattan plots were generated separately for both pKWmEB and MLM models. QQ plots were employed to identify any inflation or deflation of test statistics resulting from systemic biases such as population stratification or cryptic relatedness.

### McNemar’s test

McNemar’s test, which is a non-parametric method, is performed on matched pairs and assesses whether the proportions of a binary outcome differ significantly between the paired observations^89^. Due to being based on the chi-square test, and depending on sample numbers, McNemar’s test is generally not sensitive enough to detect small effects of variables; but it can detect major effects. For this purpose, 180 case-control matched pairs were constructed. In genotype–phenotype association studies, the greatest advantage of McNemar’s test is its ability to standardize variables such as age, gender, breed, and farm, which could directly influence the results. Thefore, matching was perform based on gender, type of birth, and maternal age. Briefly, each case was matched with a control of the same gender, the same type of birth, and the same maternal age. Thus, the parameters used in both GWA methods were also utilized in the McNemar’s test.

Each member of a case-control pair is assigned a value of ‘+’ or ‘−’ depending on whether the risk/protective factor is present (+) or absent (−) and falls into one of four possible categories pair status categories: ‘+,+’; ‘+,−’; ‘−,+’; or ‘−,−’. All subsequent test statistics are calculated from these values (Table 4). In McNemar’s test, only the discordant pairs (*b* and* c*) are informative.

**Table 4:** McNemar pair classifications in a 2 × 2 contingency table.

		Non-diarrheic		Row totals
		Genetic risk factor present	Genetic risk factor absent	
Diarrheic	Genetic risk factor present	+,+ (a)	+,− (b)	a + b
	Genetic risk factor absent	−,+ (c)	−,− (d)	c + d
		a + c	b + d	n

Statistical power for each SNP under McNemar’s test was calculated using G*Power v3.1.9.7 software (Universtat Düsseldorf, Germany). In genetic association studies, a minimum of 0.80 statistical power (*p* ≤ 0.05) is commonly employed to ensure reliable statistics^90,91^. Therefore, only the SNPs meeting a statistical power of ≥ 0.80 (*p* ≤ 0.05) were deemed to have a significantly effect on neonatal diarrhea in the McNemar’s test.

### Gene enrichment and network analysis

Gene enrichment and network analysis were performed using ShinyGO^92^, a graphical gene-set enrichment tool, employing KEGG database related to the *Ovis aries* genome. The false discovery rate (FDR) cutoff threshold for gene enrichment was set to 0.05. Visualizations were generated using the same tool.

### Ethical statement

This study was conducted in compliance with the guidelines of the Animal Experiments Local Ethics Committee and with an experimental protocol approved by the Ethics Committee for the Use of Animals in Research and Experimentation at the Sheep Breeding and Research Institute, Turkey (Approval No: 04.10.2021/049) and the authors complied with the ARRIVE guidelines.

## Data Availability

The data supporting the findings of this study are available from the corresponding author (yalcinyaman@gmail.com).

## References

[1] Dwyer C (2016). Invited review: Improving neonatal survival in small ruminants: Science into practice. Animal.

[2] Khan A, Sultan MA, Jalvi MA, Hussaın I (2006). Risk factors of lamb mortality in Pakistan. Anim. Res..

[3] Butsashvili M (2009). Risk factors of mortality in septic newborns in neonatal intensive care units (NICUs) in Tbilisi, the republic of Georgia. Eur. J. Epidemiol..

[4] Tedla M, Degefa K (2017). Bacteriological study of calf colisepticemia in alage dairy farm, Southern Ethiopia. BMC Res. Notes.

[5] Wang S, Cui D, Lv Y, Yan Z, Zhang J (2022). CANGPU oral liquid as a possible alternative to antibiotics for the control of undifferentiated calf diarrhea. Front. Vet. Sci..

[6] Ghazy AA, Abdel-Shafy S, Shaapan RM (2015). Cryptosporidiosis in animals and man: 1. Taxonomic classification, life cycle, epidemiology and zoonotic importance. Asian J. Epidemiol..

[7] Baroudi D (2018). Zoonotic cryptosporidium species and subtypes in lambs and goat kids in algeria. Parasites Vectors.

[8] Kalkanov I, Dinev I, Zarkov I (2021). Etiological and pathomorphological investigations of coronavirus and rotavirus gastroenteritis in goat kids and lambs. Maced. Vet. Rev..

[9] Zhong T (2022). Diarrhea in suckling lambs is associated with changes in gut microbiota, serum immunological and biochemical parameters in an intensive production system. Front. Microbiol..

[10] El-Shazly WSA (2020). Prevalence of multi drug resistant escherichia coli in diarrheic ruminants. Benha Vet. Med. J..

[11] Dahmani H, Ouchene N, Dahmani A, Ouchene-Khelifi NA, Oumouna M (2020). First report on cryptosporidium parvum, escherichia coli K99, rotavirus and coronavirus in neonatal lambs from north-center region, Algeria. Comp. Immunol. Microbiol. Infect. Dis..

[12] Imboden M, Schaefer DA, Bremel RD, Homan EJ, Riggs MW (2012). Antibody fusions reduce onset of experimental cryptosporidium parvum infection in calves. Vet. Parasitol..

[13] Gökçe E, Kırmızıgül AH, Erdoğan HM, Çitil M (2013). Risk factors associated with passive immunity, health, birth weight and growth performance in lambs I. Effect of parity, dam’s health, birth weight, gender, type of birth and lambing season on morbidity and mortality. Kafkas Univ. Vet. Fak. Derg..

[14] Dhama K, Chauhan RS, Mahendran M, Malik SVS (2008). Rotavirus diarrhea in bovines and other domestic animals. Vet. Res. Commun..

[15] Wu Y (2020). Genetic diversity of cryptosporidium parvum in neonatal dairy calves in Xinjiang China. Pathogens.

[16] Wei X (2021). Detection of infectious agents causing neonatal calf diarrhea on two large dairy farms in Yangxin county, Shandong province China. Front. Vet. Sci..

[17] Abdel-Moein KA, Saeed H (2016). The zoonotic potential of giardia intestinalis assemblage E in rural settings. Parasitol. Res..

[18] Horton B, Bridle H, Alexander CL, Katzer F (2018). Giardia duodenalisin the UK: Current knowledge of risk factors and public health implications. Parasitology.

[19] Vasco K, Graham JP, Trueba G (2016). Detection of zoonotic enteropathogens in children and domestic animals in a semirural community in ecuador. Appl. Environ. Microbiol..

[20] Harrison EM (2013). Whole genome sequencing identifies zoonotic transmission of MRSA isolates with the novel mecA homologue mecC. EMBO Mol. Med..

[21] Martella V, Bànyai K, Matthijnssens J, Buonavoglia C, Ciarlet M (2010). Zoonotic aspects of rotaviruses. Vet. Microbiol..

[22] Li Q (2023). Cross-species transmission, evolution and zoonotic potential of coronaviruses. Front. Cell. Infect. Microbiol..

[23] Sahana G (2014). Genome-wide association study using high-density single nucleotide polymorphism arrays and whole-genome sequences for clinical mastitis traits in dairy cattle. J. Dairy Sci..

[24] Welderufael BG, Løvendahl P, De Koning DJ, Janss LLG, Fikse WF (2018). Genome-wide association study for susceptibility to and recoverability from mastitis in danish holstein cows. Front. Genet..

[25] Kurz JP (2018). A genome-wide association study for mastitis resistance in phenotypically well-characterized holstein dairy cattle using a selective genotyping approach. Immunogenetics.

[26] Freebern E (2020). GWAS and fine-mapping of livability and six disease traits in holstein cattle. BMC Genom..

[27] May K, Sames L, Scheper C, König S (2022). Genomic loci and genetic parameters for uterine diseases in first-parity Holstein cows and associations with milk production and fertility. J. Dairy Sci..

[28] Oelschlaegel D (2022). Functional variants associated with CMPK2 and in ASB16 influence bovine digital dermatitis. Front. Genet..

[29] Bay V (2023). The bovine foot skin microbiota is associated with host genotype and the development of infectious digital dermatitis lesions. Microbiome.

[30] Abdou NEMI (2021). Cross-sectional study and genotyping of rotavirus-A infections in ruminants in kuwait. BMC Vet. Res..

[31] Caffarena RD (2021). Causes of neonatal calf diarrhea and mortality in pasture-based dairy herds in Uruguay: A farm-matched case-control study. Braz. J. Microbiol..

[32] Wen Y (2017). Methodological implementation of mixed linear models in multi-locus genome-wide association studies. Brief. Bioinform..

[33] Wang S (2016). Improving power and accuracy of genome-wide association studies via a multi-locus mixed linear model methodology. Sci. Rep..

[34] Cui Y, Zhang F, Zhou Y (2018). The application of multi-locus GWAS for the detection of salt-tolerance LOCI in rice. Front. Plant Sci..

[35] Nakata R (2020). Slco2a1 deficiency exacerbates experimental colitis via inflammasome activation in macrophages: A possible mechanism of chronic enteropathy associated with SLCO2A1 gene. Sci. Rep..

[36] Yao C, Narumiya S (2018). Prostaglandin-cytokine crosstalk in chronic inflammation. Br. J. Pharmacol..

[37] Song W, Li D, Tao L, Luo Q, Chen L (2020). Solute carrier transporters: The metabolic gatekeepers of immune cells. Acta Pharm. Sin. B.

[38] Maltas J, Reed H, Porter A, Malliri A (2020). Mechanisms and consequences of dysregulation of the tiam family of Rac activators in disease. Biochem. Soc. Trans..

[39] Diamantopoulou Z (2017). TIAM1 antagonizes TAZ/YAP both in the destruction complex in the cytoplasm and in the nucleus to inhibit invasion of intestinal epithelial cells. Cancer Cell.

[40] Veluthakal R, Madathilparambil S, McDonald P, Olson LK, Kowluru A (2009). Regulatory roles for Tiam1, a guanine nucleotide exchange factor for Rac1, in glucose-stimulated insulin secretion in pancreatic β-cells. Biochem. Pharmacol..

[41] Kim SW (2017). Identification of genetic susceptibility loci for intestinal Behçet’s disease. Sci. Rep..

[42] Burgner D (2009). A genome-wide association study identifies novel and functionally related susceptibility LOCI for Kawasaki disease. PLoS Genet..

[43] Hong T (2021). Epstein-Barr virus nuclear antigen 2 extensively rewires the human chromatin landscape at autoimmune risk loci. Genome Res..

[44] Pierzchała M (2020). Identification of differentially expressed gene transcripts in porcine endometrium during early stages of pregnancy. Life.

[45] Bapat J (2023). CASC4/GOLM2 drives high grade serous carcinoma anoikis resistance through the recycling of EGFR. Cancer Gene Ther..

[46] Wang L (2023). Plasma proteomics of SARS-CoV-2 infection and severity reveals impact on Alzheimer’s and coronary disease pathways. iScience.

[47] Urbiola-Salvador V, De Souza SL, Grešner P, Qureshi T, Chen Z (2023). Plasma proteomics unveil novel immune signatures and biomarkers upon SARS-CoV-2 infection. Int. J. Mol. Sci..

[48] Bonomi A (2020). Analysis of the genetic variants associated with circulating levels of sgp130. Results from the IMPROVE study. Genes Immun..

[49] Li Q (2009). Microarray analysis of lymphatic tissue reveals stage-specific, gene expression signatures in HIV-1 infection. J. Immunol..

[50] Byadgi O (2021). Innate immune-gene expression during experimental amyloodiniosis in European seabass (*Dicentrarchus labrax*). Vet. Immunol. Immunopathol..

[51] Marchbank KJ, Kulik L, Gipson MG, Morgan BP, Holers VM (2002). Expression of human complement receptor type 2 (CD21) in mice during early B cell development results in a reduction in mature B cells and hypogammaglobulinemia. J. Immunol..

[52] Boackle SA (2004). CR1/CR2 deficiency alters IGG3 autoantibody production and IGA glomerular deposition in the MRL/LPRModel of SLE. Autoimmunity.

[53] Asokan R, Banda NK, Szakonyi G, Chen XS, Holers VM (2013). Human complement receptor 2 (CR2/CD21) as a receptor for DNA: Implications for its roles in the immune response and the pathogenesis of systemic lupus erythematosus (SLE). Mol. Immunol..

[54] Ewbank, J.B. Mycobacterium tuberculosis induced transcription in macrophages: The role of TPL2/ERK signalling in the negative regulation of type I interferon production and implications for control of tuberculosis. Thesis, University College London for the Degree of Doctor of Philosophy (2012).

[55] Dupnik K (2014). Transcriptional changes that characterize the immune reactions of leprosy. J. Infect. Dis..

[56] Ghandikota S, Sharma M, Jegga AG (2021). Secondary analysis of transcriptomes of SARS-CoV-2 infection models to characterize COVID-19. Patterns.

[57] Hertel L, Mocarski ES (2004). Global analysis of host cell gene expression late during cytomegalovirus infection reveals extensive dysregulation of cell cycle gene expression and induction of pseudomitosis independent of US28 function. J. Virol..

[58] Gopalakrishnan J (2021). Variants on the UBE2L3/YDJC autoimmune disease risk haplotype increase UBE2L3 expression by modulating CCCTC-binding factor and YY1 binding. Arthr. Rheumatol..

[59] Quinn EM (2015). Transcriptome analysis of CD4+ T cells in coeliac disease reveals imprint of BACH2 and IFNΓ regulation. PLoS ONE.

[60] Rothwell S (2015). Dense genotyping of immune-related loci in idiopathic inflammatory myopathies confirms HLA alleles as the strongest genetic risk factor and suggests different genetic background for major clinical subgroups. Ann. Rheum. Dis..

[61] Lassen KG (2016). Genetic coding variant in GPR65 alters lysosomal pH and links lysosomal dysfunction with colitis risk. Immunity.

[62] Zhou S, Shu Y (2022). Transcriptional regulation of solute carrier drug transporters. Drug Metab. Dispos..

[63] Xu K (2022). SLC22A8: An indicator for tumor immune microenvironment and prognosis of ccRCC from a comprehensive analysis of bioinformatics. Medicine.

[64] Akanuma SI (2011). Attenuation of prostaglandin E2 elimination across the mouse blood-brain barrier in lipopolysaccharide-induced inflammation and additive inhibitory effect of cefmetazole. Fluids Barriers CNS.

[65] Tolar P, Sohn HS, Pierce SK (2008). Viewing the antigen-induced initiation of B-cell activation in living cells. Immunol. Rev..

[66] Kim YM (2006). Monovalent ligation of the B cell receptor induces receptor activation but fails to promote antigen presentation. Proc. Natl. Acad. Sci. USA.

[67] Matsubara N (2018). CD22-binding synthetic sialosides regulate B lymphocyte proliferation through CD22 ligand-dependent and independent pathways, and enhance antibody production in mice. Front. Immunol..

[68] Alhakeem SS (2015). Role of B cell receptor signaling in IL-10 production by normal and malignant B-1 cells. Ann. New York Acad. Sci..

[69] Schneider MC (2009). Neisseria meningitidis recruits factor H using protein mimicry of host carbohydrates. Nature.

[70] Dunkelberger J, Song W (2009). Complement and its role in innate and adaptive immune responses. Cell Res..

[71] Zarantonello A, Pedersen H, Laursen NS, Andersen GR (2021). Nanobodies provide insight into the molecular mechanisms of the complement cascade and offer new therapeutic strategies. Biomolecules.

[72] Fujita T (2002). Evolution of the lectin–complement pathway and its role in innate immunity. Nat. Rev. Immunol..

[73] Rus H, Cudrici C, Niculescu F (2005). The role of the complement system in innate immunity. Immunol. Res..

[74] Palta S, Saroa R, Palta A (2014). Overview of the coagulation system. Indian J. Anaesth..

[75] Haynes LM, Orfeo T, Mann KG, Everse SJ, Brummel-Ziedins KE (2017). Probing the dynamics of clot-bound thrombin at venous shear rates. Biophys. J..

[76] Soh UJK, Dores MR, Chen B, Trejo J (2010). Signal transduction by protease-activated receptors. Br. J. Pharmacol..

[77] Moreau ME (2005). The kallikrein-kinin system: Current and future pharmacological targets. J. Pharmacol. Sci..

[78] Seita J, Weissman IL (2010). Hematopoietic stem cell: Self-renewal versus differentiation. WIREs Mech. Dis..

[79] Woolthuis CM, Park CY (2016). Hematopoietic stem/progenitor cell commitment to the megakaryocyte lineage. Blood.

[80] Boyer JL, Soroka CJ (2021). Bile formation and secretion: An update. J. Hepatol..

[81] Forker EL (1967). Two sites of bile formation as determined by mannitol and erythritol clearance in the guinea pig. J. Clin. Invest..

[82] Zhuang S, Li Q, Cai L, Wang C, Lei X (2017). Chemoproteomic profiling of bile acid interacting proteins. ACS Cent. Sci..

[83] Purcell S (2007). PLINK: A tool set for whole-genome association and population-based linkage analyses. Am. J. Hum. Genet..

[84] Bradbury PJ (2007). TASSEL: Software for association mapping of complex traits in diverse samples. Bioinformatics.

[85] Zhang Y (2020). MrMLM V4.0.2: An R platform for multi-locus genome-wide association studies. Genom. Proteom. Bioinform..

[86] Ren W, Wen Y, Dunwell JM, Zhang YM (2017). pKWmEB: Integration of Kruskal-Wallis test with empirical bayes under polygenic background control for multi-locus genome-wide association study. Heredity.

[87] Tamba CL, Ni Y, Zhang Y (2017). Iterative sure independence screening EM-bayesian LASSO algorithm for multi-locus genome-wide association studies. PLoS Comput. Biol..

[88] Zhou X, Stephens M (2012). Genome-wide efficient mixed-model analysis for association studies. Nat. Genet..

[89] McNemar Q (1947). Note on the sampling error of the difference between correlated proportions or percentages. Psychometrika.

[90] Ahn C (2006). Sample size and power estimation in case-control genetic association studies. Genom. Inform..

[91] Hong EP, Park JW (2012). Sample size and statistical power calculation in genetic association studies. Genom. Inform..

[92] Ge X, Jung D, Yao R (2019). ShinyGO: A graphical gene-set enrichment tool for animals and plants. Bioinformatics.

